# Rewinding the Ratchet: Rare Recombination Locally Rescues Neo-W Degeneration and Generates Plateaus of Sex-Chromosome Divergence

**DOI:** 10.1093/molbev/msae124

**Published:** 2024-07-01

**Authors:** Thomas Decroly, Roger Vila, Konrad Lohse, Alexander Mackintosh

**Affiliations:** Institute of Ecology and Evolution, University of Edinburgh, Edinburgh EH9 3FL, UK; Institut de Biologia Evolutiva (CSIC-Universitat Pompeu Fabra), Passeig Marítim de la Barceloneta 37, ESP-08003 Barcelona, Spain; Institute of Ecology and Evolution, University of Edinburgh, Edinburgh EH9 3FL, UK; Institute of Ecology and Evolution, University of Edinburgh, Edinburgh EH9 3FL, UK

**Keywords:** neo-sex chromosome, recombination, Muller’s ratchet, Hill–Robertson interference

## Abstract

Natural selection is less efficient in the absence of recombination. As a result, nonrecombining sequences, such as sex chromosomes, tend to degenerate over time. Although the outcomes of recombination arrest are typically observed after many millions of generations, recent neo-sex chromosomes can give insight into the early stages of this process. Here, we investigate the evolution of neo-sex chromosomes in the Spanish marbled white butterfly, *Melanargia ines*, where a Z-autosome fusion has turned the homologous autosome into a nonrecombining neo-W chromosome. We show that these neo-sex chromosomes are likely limited to the Iberian population of *M. ines*, and that they arose around the time when this population split from North-African populations, around 1.5 million years ago. Recombination arrest of the neo-W chromosome has led to an excess of premature stop-codons and frame-shift mutations, and reduced gene expression compared to the neo-Z chromosome. Surprisingly, we identified two regions of ∼1 Mb at one end of the neo-W that are both less diverged from the neo-Z and less degraded than the rest of the chromosome, suggesting a history of rare but repeated genetic exchange between the two neo-sex chromosomes. These plateaus of neo-sex chromosome divergence suggest that neo-W degradation can be locally reversed by rare recombination between neo-W and neo-Z chromosomes.

## Introduction

Recombination allows alleles to move between genetic backgrounds. This results in more efficient natural selection, as the fitness effect of a new allele is decoupled from the background on which it arose (Fisher 1930). A reduction in the rate of recombination, therefore, results in less efficient purging of deleterious and reduced fixation of beneficial alleles (Muller 1964; Hill and Robertson 1968). While recombination is often considerably reduced in particular regions of the genome, e.g. nearby centromeres (Choo 1998), it can also be suppressed entirely on certain chromosomes. Sex-limited chromosomes may only recombine at a particular region (e.g. the pseudo-autosomal region of the mammalian Y chromosome) or not at all (e.g. Lepidopteran W chromosomes). Nonrecombining sex chromosomes typically exhibit signs of sequence degeneration such as elevated rates of loss-of-function mutations, sequence loss, transposable element proliferation, and pseudogenization of genes (Bachtrog 2013). Genomic analyses of nonrecombining sex chromosomes show that recombination arrest leads to natural selection becoming less efficient, but it is less clear exactly which evolutionary processes (e.g. Muller’s ratchet, hitchhiking of deleterious mutations, background selection) are most important in this process (Charlesworth and Charlesworth 2000; Bachtrog 2008) and to what extent escape from degeneration is possible. There are also unresolved questions about how nonrecombining chromosomes affect gene expression, and, ultimately, phenotypes. For example, it is unclear whether the low levels of gene expression observed on some nonrecombining chromosomes come about gradually as a result of sequence degeneration, or if instead functional genes are silenced first, allowing for the neutral accumulation of loss-of-function mutations (Lenormand et al. 2020). Resolving these questions requires both careful inference from natural examples of recombination arrest and population genetic modeling of the relevant processes (Bachtrog 2008).

Neo-sex chromosomes that form as a result of a fusion between an autosome and a sex chromosome have been reported in a wide variety of taxa (Howell et al. 2009; Gil-Fernández et al. 2020; Huang et al. 2022; Akagi et al. 2023; Sacchi et al. 2024). Sex-autosome linkage means that the previously autosomal chromosome will follow a sex-specific pattern of inheritance. Consequently, in species with achiasmatic meiosis (where recombination only happens in the homogametic sex) the evolution of neo-sex chromosomes leads to recombination suppression. In *Drosophila*, where meiosis in males is typically achiasmatic, a neo-Y chromosome will experience a sudden arrest of recombination. The same is also true for neo-W chromosomes in Lepidoptera, as female meiosis is achiasmatic. Recombination arrest can also be generated by X-autosome and Z-autosome fusions, as the unfused autosome will cosegregate with the Y/W chromosome and becomes a nonrecombining neo-Y/neo-W chromosome. Recent sex-autosome chromosome fusions in achiasmatic species can thus provide insight into the effect of recombination suppression (Charlesworth and Charlesworth 2000; Wei and Bachtrog 2019).

High rates of chromosome fusion have been observed in certain Lepidopteran lineages (Gauthier et al. 2022; Höök et al. 2023; Mackintosh et al. 2023). This may be partly explained by the fact that Lepidoptera have holocentric chromosomes with diffuse centromeres, which is predicted to facilitate proper pairing and segregation of multivalents during meiosis (Lucek et al. 2022; Senaratne et al. 2022). The Lepidopteran Z chromosome is involved in fusions more often than any autosome (Wright et al. 2024), leading to a high rate of sex chromosome evolution. While many of the neo-sex chromosomes known in Lepidoptera are old (i.e. shared by multiple genera), young neo-sex chromosomes have been described in a handful of taxa (Smith et al. 2016; Mongue et al. 2017; Mackintosh et al. 2022; Berner et al. 2023; Höök et al. 2023; Rueda-M et al. 2023). Some of these neo-sex chromosomes are nonetheless highly diverged, meaning that little can be inferred about the early stages of neo-W degeneration (Mongue et al. 2017). Others, however, are so young that little degeneration is observed at all (Martin et al. 2020). Recently, neo-sex chromosomes have been identified in the *sara*/*sapho* clade of *Heliconius* (Rueda-M et al. 2023) as well as in the pierid butterfly *Leptidea sinapis* (Höök et al. 2023), with both cases involving step-wise fusions of autosomes to sex chromosomes. Importantly, the neo-sex chromosomes in these taxa are old enough to have accumulated some divergence but young enough to provide insight into the consequences of recombination arrest. Identification and analysis of natural systems such as these provides an opportunity to better understand how nonrecombining sex chromosomes evolve over time.

The nymphalid butterfly *Melanargia ines* (the Spanish marbled white) is found on the Iberian Peninsula and the Maghreb. A previous analysis of mitochondrial sequence data revealed a deep split between Iberian and North-African populations, as well as a more recent split between Western and Eastern regions of the Maghreb (Dapporto et al. 2022). Additionally, the karyotype of *M. ines* has been reported as n=13 in males (de Lesse 1970), which is greatly reduced compared to the ancestral karyotype of n=31 of most Nymphalidae butterflies and suggests a recent history of multiple chromosome fusions. Here, we report the discovery of a Z-autosome fusion in the Iberian population of *M. ines*. These neo-sex chromosomes are intermediate in age, and so, represent a promising system to characterize the mechanisms of sequence degeneration due to recombination arrest. We generate a chromosome-level genome assembly and analyse whole-genome resequencing (WGS) data to address the following questions:

- What is the distribution of the neo-sex chromosomes across *M. ines* populations?
- What is the age and evolutionary history of the neo-sex chromosomes and how does it relate to the population history of *M. ines*?
- How complete is recombination arrest of the neo-W chromosome?
- How degenerated is the neo-W chromosome?

## Results

### A Genome Assembly of *Melanargia ines*

**Fig. 1.**
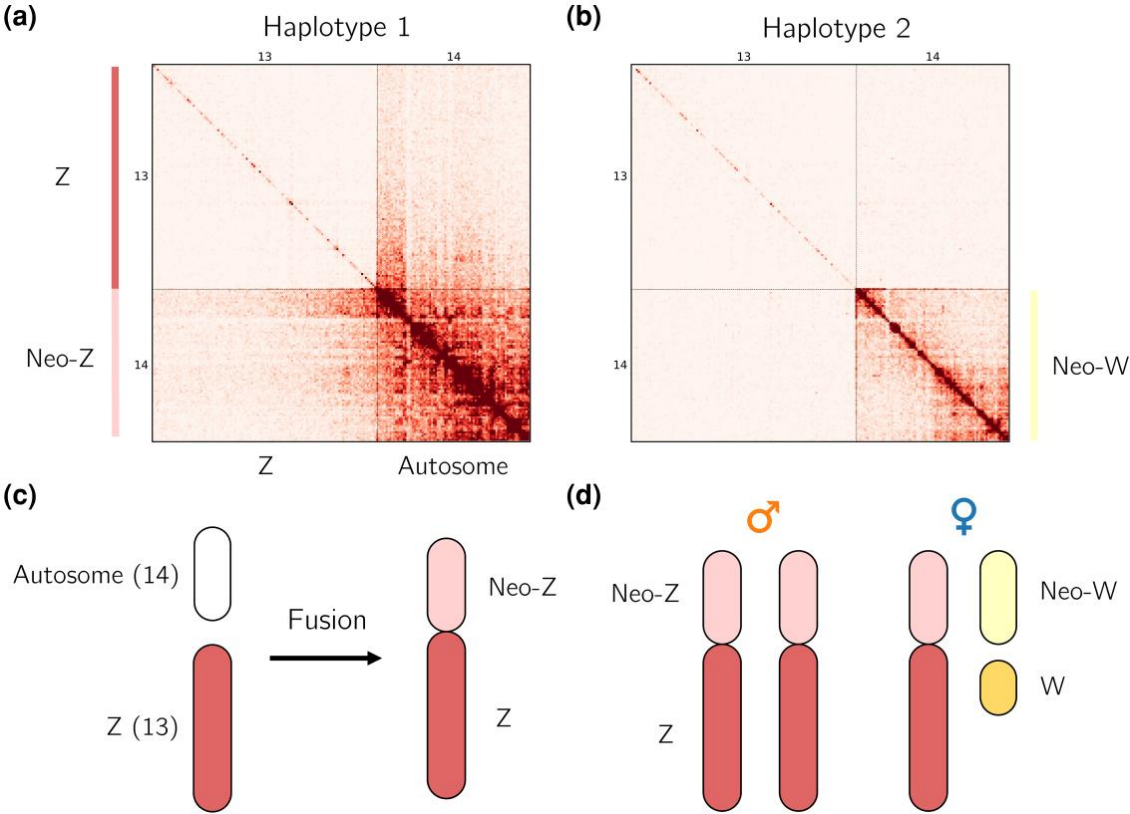
Z-autosome fusion in *M. ines*. a) The Hi-C map of chromosome 13 (Z) and chromosome 14 for the first haplotype reveals interchromosomal contacts indicative of a Z-autosome fusion. b) These are absent in the second haplotype. c) Schematic of the fusion between chromosome 14 and the Z chromosome and d) the neo-sex chromosome karyotype. When comparing the neo-Z and neo-W, we refer to the neo-Z as the segment homologous to the neo-W.

We generated a chromosome-level genome assembly for *M. ines* using a combination of Pacbio long-read, Illumina short-read and HiC data generated from two individuals sampled in Portugal (PacBio and Illumina: male PT_MI_8 and HiC: female PT_MI_86; supplementary table S1, Supplementary Material online). The assembly is 421.7 Mb in length, with a contig N50 of 4.3 Mb. The assembly contains 14 chromosome-level sequences (hereafter simply referred to as chromosomes) which range from 13.0 to 51.4 Mb in length. In contrast, de Lesse (1970) only observed 13 chromosomes in the spermatocytes of a male *M. ines* individual from Spain. We further investigated the karyotype of *M. ines* by constructing haplotype-specific HiC maps as described in Mackintosh et al. (2022). These HiC maps, which were generated from a female sample, contain very few read pairs that map exclusively to chromosome 13 (supplementary table S2, Supplementary Material online). This is consistent with chromosome 13 being the Z chromosome, as this would be present in a single copy in females and so cannot be phased into haplotypes (we also confirm this result using sex-specific read coverage; supplementary table S3, Supplementary Material online). We find that chromosomes 13 and 14 display a high density of HiC contacts in 1 haplotype, but not the other (Fig. 1). These results are consistent with this female individual possessing a Z-autosome fusion as well as another (unfused) copy of the same autosome. Given that the W chromosome is absent from our genome assembly (contigs were assembled from male-derived reads), we cannot test whether the other copy of chromosome 14 is fused to the W. However, we expect this chromosome to behave as a neo-W irrespective of physical linkage to the ancestral W given that meiosis is achiasmatic in females.

A genome alignment between *M. ines* and the congener *M. galathea* (Vila et al. 2022) shows that chromosomes 13 and 14 were unfused in their common ancestor. This supports a single Z-autosome fusion without subsequent rearrangements (supplementary fig. S1, Supplementary Material online). Hereafter, we refer to chromosome 13 as the Z and chromosome 14 as the neo-sex chromosome. When referring to a specific homolog, we use the terms neo-Z (which is fused to the Z) and neo-W (which is W linked). Assuming that the Z-autosome fusion is at high frequency, we would expect males to have 2n=26 chromosomes (12 pairs of autosomes and the Z-autosome fusion), whereas females should have 2n=27 chromosomes (12 pairs of autosomes, 1 copy of the Z-autosome fusion, the ancestral W and the neo-W). Our data are, therefore, consistent with the observations of de Lesse (1970) and suggests that this neo-sex chromosome system is young given that neo-Z and neo-W reads both map to the neo-Z reference sequence.

### Population Structure

**Fig. 2.**
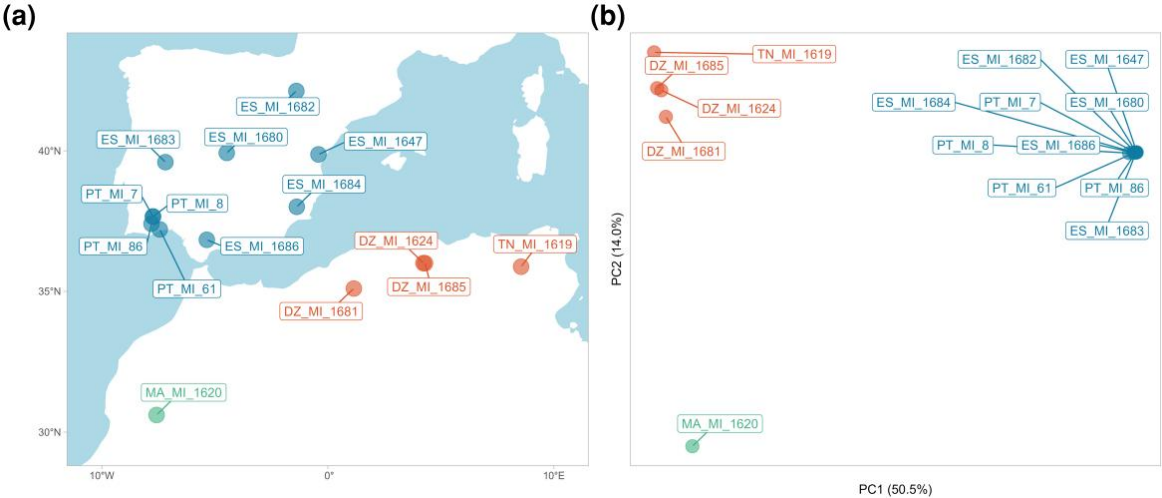
a) The *M. ines* WGS samples include 10 individuals from Iberia, and five from the Maghreb. Samples are colored according to PCA clusters. b) Iberian, Western and Eastern Maghreb samples form three distinct clusters in a PCA: PC1 separates Iberia and North-Africa, PC2 separates Eastern and Western Maghreb samples. Sample labels reflect the standard ISO 2 letters codes.

We generated WGS data for a total of 15 *M. ines* butterflies from Iberia and the Maghreb (Fig. 2a, supplementary table S1, Supplementary Material online). A principal component analysis revealed three distinct population clusters: PC1 separated samples from the Iberian Peninsula and the Maghreb (51% of the variance explained), while PC2 separated Western and Eastern populations of the Maghreb (Fig. 2b). Concordant with the PCA, substantial genetic structure was observed between North-African and European populations of *M. ines* ($F_{\mathrm{ST}}=0.38$, $d_{xy}=0.032$), indicating a deep split between the continents. Western and Eastern populations of the Maghreb also showed evidence of population structure ($F_{\mathrm{ST}}=0.20$, $d_{xy}=0.025$). Nucleotide diversity differed among the three populations, being highest in Eastern Maghreb ($\pi_{4D}=0.021$), intermediate in Western Maghreb ($\pi_{4D}=0.013$), and lowest in Iberia ($\pi_{4D}=0.008$; Table 1).

**Table 1:** Nucleotide diversity at 4- and 0-fold degenerate sites for autosomes, the neo-sex chromosomes and the Z chromosome

Autosomes	$\pi_{4D}$	$\pi_{0D}$
Iberia	0.0075	0.0016
Eastern Maghreb	0.020	0.0032
Western Maghreb	0.013	0.0021

Neo-sex chromosome		
Neo-Z	0.0072	0.0019
Neo-W	0.00077	0.00027
Eastern Maghreb	0.017	0.0033
Western Maghreb	0.012	0.0027

Z chromosome		
Iberia	0.0052	0.0012
Eastern Maghreb	0.018	0.0024
Western Maghreb	0.010	0.0015

### Neo-Sex Chromosome Distribution and Frequency

The haplotype-specific HiC maps show the presence of a Z-autosome fusion in one female individual from Portugal. Large structural variants, such as chromosome fusions and inversions, are difficult to detect using short-read sequencing data. However, the complete shutdown of recombination between the neo-W and neo-Z leads to predictable patterns in WGS data. First, given that neo-W reads still map to the neo-Z reference sequence, the density of heterozygous sites on the neo-sex chromosome is expected to be significantly higher in heterogametic females (ZW) compared to homogametic males (ZZ), as heterozygosity in females will reflect the divergence between the neo-sex chromosomes. Second, given diverged, female-specific, neo-W chromosomes, the genetic structure of the neo-sex chromosome should reflect the sex rather than the geography of samples. In other words, genetic variation is expected to cluster by sex for the neo-sex chromosome, with females being genetically closer to each other than to males, and vice versa. Neither pattern is expected for autosomes, which are recombining and not sex limited.

**Fig. 3.**
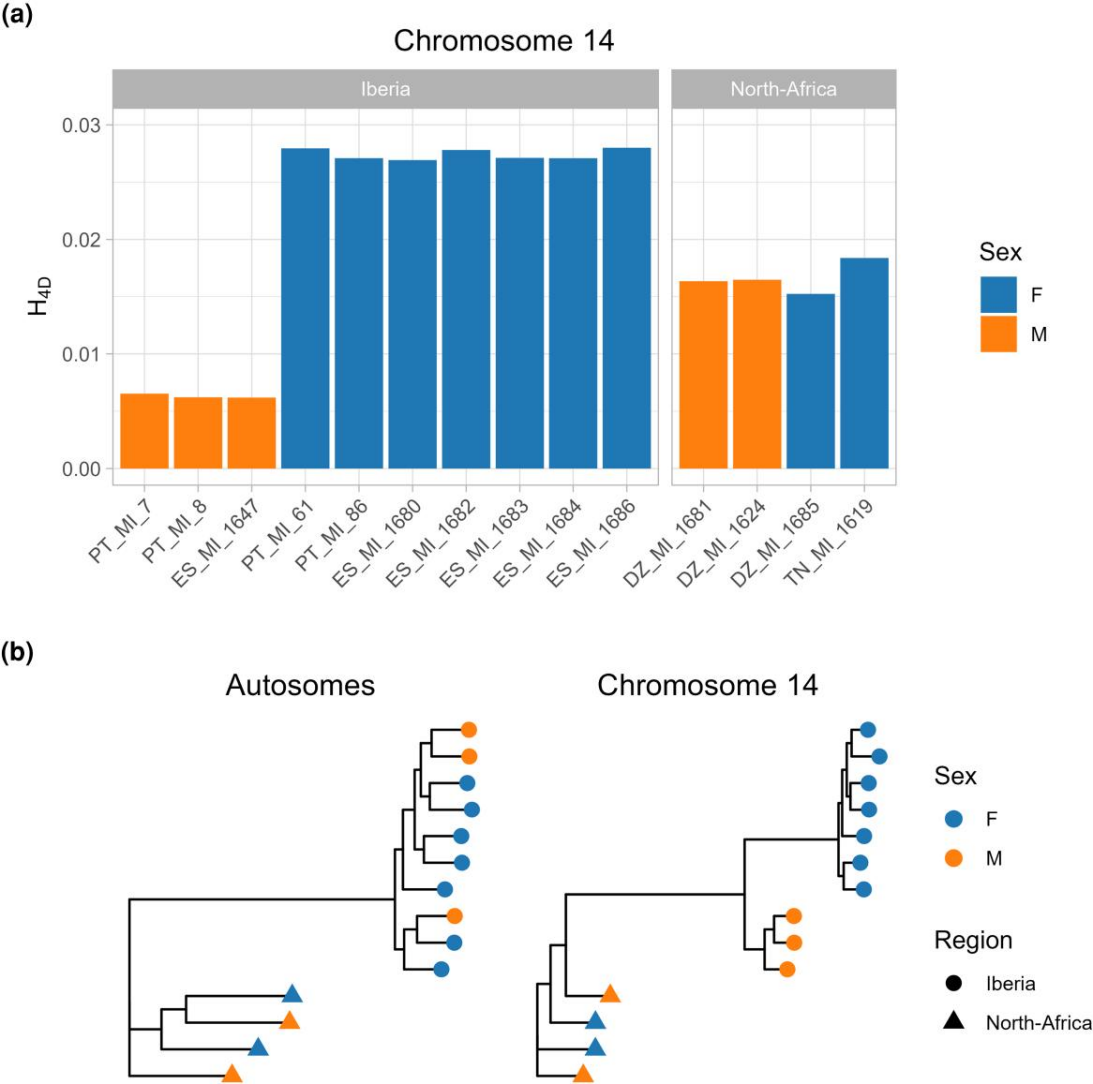
Detection of the neo-sex chromosome. a) Per-site heterozygosity ($H_{4D}$) on chromosome 14 across Iberian samples is substantially larger in Iberian females than in Iberian males. By contrast, heterozygosity is similar across Eastern Maghreb samples, irrespective of sex. b) Maximum likelihood phylogenetic trees for the autosomes and chromosome 14. Clustering by sex is observed for chromosome 14, but only for Iberian samples.

Focusing on the neo-sex chromosomes, Iberian females show a high density of heterozygous sites at 4-fold degenerate (i.e. putatively neutral) codon positions ($H_{4D}$ = 0.027). In contrast, in males $H_{4D}$ is significantly lower (0.006) and similar to autosomal $H_{4D}$ (∼0.006; Fig. 3). This is consistent with Iberian females possessing diverged neo-Z and neo-W chromosomes (i.e. $H_{4D}$ in females reflects the divergence between the neo-Z and neo-W), while males possess two copies of the neo-Z chromosome.

A PCA of variation for chromosome 14 suggests strong clustering by sex: PC1 separates Iberian samples by sex and explains 49.1% of the total variance (supplementary fig. S2, Supplementary Material online). In contrast, the structuring of autosomal variation for Iberian samples shows no correspondence with sex but likely reflects geography (supplementary fig. S2, Supplementary Material online). Similarly, a phylogenetic tree constructed from autosomal sequences reflects geography, whereas the phylogeny for chromosome 14 is structured by sex (Fig. 3). These patterns show that the neo-W chromosome is present in all Iberian females sampled. Assuming binomial sampling, the 95% confidence interval for the population frequency of the neo-W in Iberia is 0.59−1.

In contrast, in Eastern Maghreb samples, $H_{4D}$ on chromosome 14 is similar for males and females. Additionally, genetic structure for chromosome 14 is similar to that of other autosomes and does not cluster by sex (Fig. 3). These results lead us to conclude that the neo-sex chromosomes are absent from the Maghreb and therefore confined to Iberia.

### Age of the Z-autosome Fusion

To determine whether the neo-sex chromosomes originated before or after the split between the Iberian and Maghreb populations, we modeled both the divergence history of *M. ines* populations and the neo-Z and the neo-W chromosomes using the block-wise composite likelihood inference implemented in gIMble (Laetsch et al. 2023). Given the genetic structure between Western and Eastern Maghreb samples, the single Western Maghreb individual was removed from the analysis. We fitted three different models to the autosomal data to infer the history of divergence between the Iberian and the Eastern Maghreb populations: (i) Strict divergence between Iberia and Eastern Maghreb (DIV), i.e. no postdivergence gene flow, (ii) an isolation with migration (IM) model with a constant rate of migration from Iberia to Eastern Maghreb (${\mathrm{IM}}_{\to \mathrm{EM}}$) or in the opposite direction (${\mathrm{IM}}_{\to \mathrm{IP}}$), (iii) a migration-only model, with migration in either direction (${\mathrm{MIG}}_{\to \mathrm{EM}}$ or ${\mathrm{MIG}}_{\to \mathrm{IP}}$) without assuming a common ancestral population.

The strict divergence model (DIV) best fit the data, i.e. IM models converge to the DIV model ($m_{e}=0$) and MIG models are less well supported (supplementary table S4, Supplementary Material online). Under the DIV model we infer a split between Eastern Maghreb and Iberia (Table 2) at time $T\approx 1.5\times 10^{6}$ generations ago. Note that since *M. ines* is univoltine, time estimates in generation and years are equivalent.

**Table 2:** Maximum composite likelihood estimates of divergence and $N_{e}$ parameters under the DIV model for the three plateaus on the neo-sex chromosome, and the split between Iberian Peninsula and Eastern Maghreb populations

	${Ne}_{Neo-W}$	${Ne}_{Neo-Z}$	${Ne}_{Anc}$	*T*	lnCL	$d_{xy}$ (intergenic)
Plateau 1	$2.1\times 10^{4}$	$2.7\times 10^{5}$	$1.3\times 10^{6}$	$1.4\times 10^{6}$	− 958,297	0.022
Plateau 2	$2.1\times 10^{4}$	$2.6\times 10^{5}$	$9.8\times 10^{5}$	$8.6\times 10^{5}$	− 130,194	0.015
Plateau 3	$2.1\times 10^{4}$	$2.4\times 10^{5}$	$5.7\times 10^{5}$	$5.0\times 10^{5}$	− 31,886	0.008
	${Ne}_{Iberia}$	${Ne}_{Eastern\;Maghreb}$	${Ne}_{Anc}$	***T***	lnCL	$d_{xy}$ (intergenic)
Autosomes	$3.6\times 10^{5}$	$1.2\times 10^{6}$	$1.1\times 10^{6}$	$1.5\times 10^{6}$	− 303,465,712	0.022

We also fitted a model of strict divergence between the neo-Z and the neo-W, excluding the last 2.5 Mb of the neo-sex chromosomes (see next section). The population divergence time, which should reflect the onset of recombination arrest, was estimated at $1.4\times 10^{6}$ generations ago. This estimate for the origin of the neo-sex chromosomes is slightly more recent than the inferred split between Maghreb and Iberian populations which is compatible with the neo-sex chromosomes arising in Iberia. However, given the large confidence intervals of time estimates (in particular those for the neo-sex chromosomes divergence), we do not have power to determine whether the origin of the neo-sex chromosome pre- or postdated the split between Iberian and North-African populations (supplementary fig. S4, Supplementary Material online)

### Plateaus of Divergence Suggest Historical Recombination Events between the Neo-Z and Neo-W Chromosomes

**Fig. 4.**
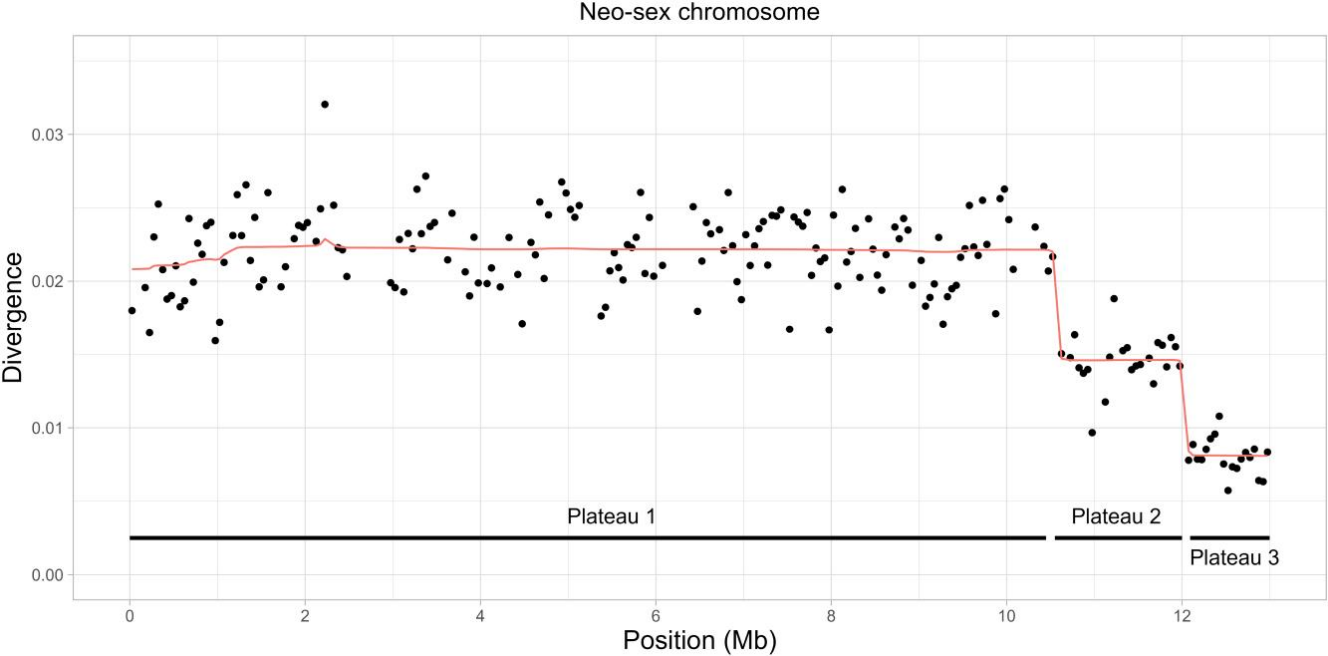
The genetic divergence between the neo-sex chromosomes (as measured by *H*) shows three distinct plateaus. The line represents the posterior mean divergence from a change point detection analysis. Reduced divergence between the neo-W and the neo-Z chromosome is observed for the last 2 plateaus (2.5 Mb at the end of the chromosome).

Since the neo-W is inherited as a single nonrecombining haplotype, we would expect divergence between the neo-Z and the neo-W to be relatively uniform along the neo-sex chromosome (except for local variation in mutation rate and coalescence time). Surprisingly, a change point analysis of windowed divergence between neo-Z and neo-W sequences as measured by the density of heterozygous sites (*H*) in Iberian females revealed three distinct plateaus of divergence (Fig. 4). The two plateaus exhibiting reduced divergence encompass the last 2.5 Mb of the neo-sex chromosome. These plateaus of neo-sex chromosome divergence were observed across all Iberian females, i.e. individual *H* landscapes all show the same three plateaus (supplementary fig. S3, Supplementary Material online).

The largest plateau, which encompasses the first 10.5 Mb of the chromosome, shows the greatest divergence between neo-Z and neo-W sequences and likely reflects the timing of the Z-autosome fusion (see previous section). The last two plateaus show reduced divergence between the neo-sex chromosomes, suggesting a history of recombination between the neo-W and the neo-Z. Such a scenario is surprising given that meiosis in female Lepidoptera is achiasmatic. However, rare crossover events are the most plausible explanation given the length of the plateaus (>1 Mb), and the fact that they are separated by sharp boundaries.

Repeating the demographic analysis in the previous section, but focusing on plateaus 2 and 3, allows for an estimate of the timing of recombination events. The maximum composite likelihood estimates of *T* for plateaus 2 and 3 were $8.6\times 10^{5}$ and $5.0\times 10^{5}$ generations ago, respectively (Table 2).

### Patterns of Sequence Polymorphism on the Neo-W

**Fig. 5.**
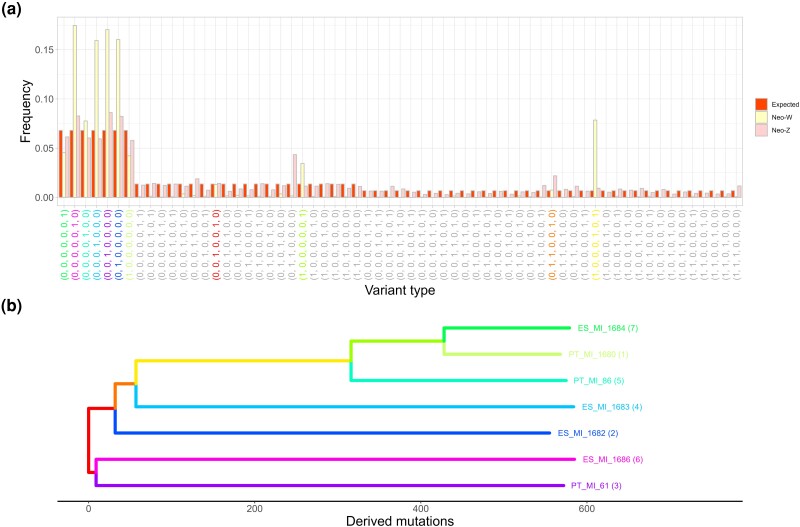
a) The folded variant type spectrum of the neo-Z and neo-W chromosomes and the corresponding expectation under the standard neutral coalescent. Variant types are colored according to their corresponding branches in the neo-W tree shown in b).

Given the evidence for recombination between the neo-Z and neo-W, we tested whether genetic diversity on the neo-W is consistent with a single tree, i.e. we asked whether there is any evidence for recent recombination events that are undetectable using windowed neo-W neo-Z divergence. Assuming that the neo-W chromosomes share a common ancestor more recently than the last recombination event between the neo-W and neo-Z, we expect neo-W variation to be consistent with a single genealogy. In line with this expectation, we find that 96% (3240 out of 3362) of the phased variants on the neo-W are compatible with a single tree, assuming an infinite sites mutation model and maximum parsimony. The 11 most common mutation types on the neo-W correspond to branches in the majority tree (Fig. 5). Moreover, the small fraction of parsimony informative variants that are inconsistent with the majority topology do not share a particular topology and are not physically clustered (as would be expected for local genealogies generated by recombination events). This suggests that these are likely the result of back-mutations and genotyping errors, rather than recombination events.

The frequency of neo-W polymorphisms which reflects a single tree is in stark contrast with the diversity among the (recombining) neo-Z haplotypes, which includes every possible type of variant. Models of population history that assume no population structure predict equal frequencies of all variants in a particular SFS class. We find that the mutation spectrum of the neo-Z haplotypes sampled from the seven Iberian females fits this simple expectation surprisingly well (Fig. 5).

In the absence of natural selection and male biased mutation rate, and assuming an equal sex ratio, neo-W diversity is expected to be one-fourth of autosomal diversity. We find that neutral site diversity among *M. ines* neo-W chromosomes is extremely low ($\pi_{4D}=0.0008$) and only $∼\frac{1}{10}$th of autosomal diversity ($\pi_{4D}=0.008$). This result is unsurprising given that natural selection will remove diversity across the entire length of this nonrecombining chromosome. While the high proportion of singleton mutations among neo-W chromosomes (corresponding to external branches; Fig. 5b) is compatible with a past selective sweep, it is difficult to glean the selective history of a nonrecombining chromosome given that there is only a single genealogy (see Discussion).

**Fig. 6.**
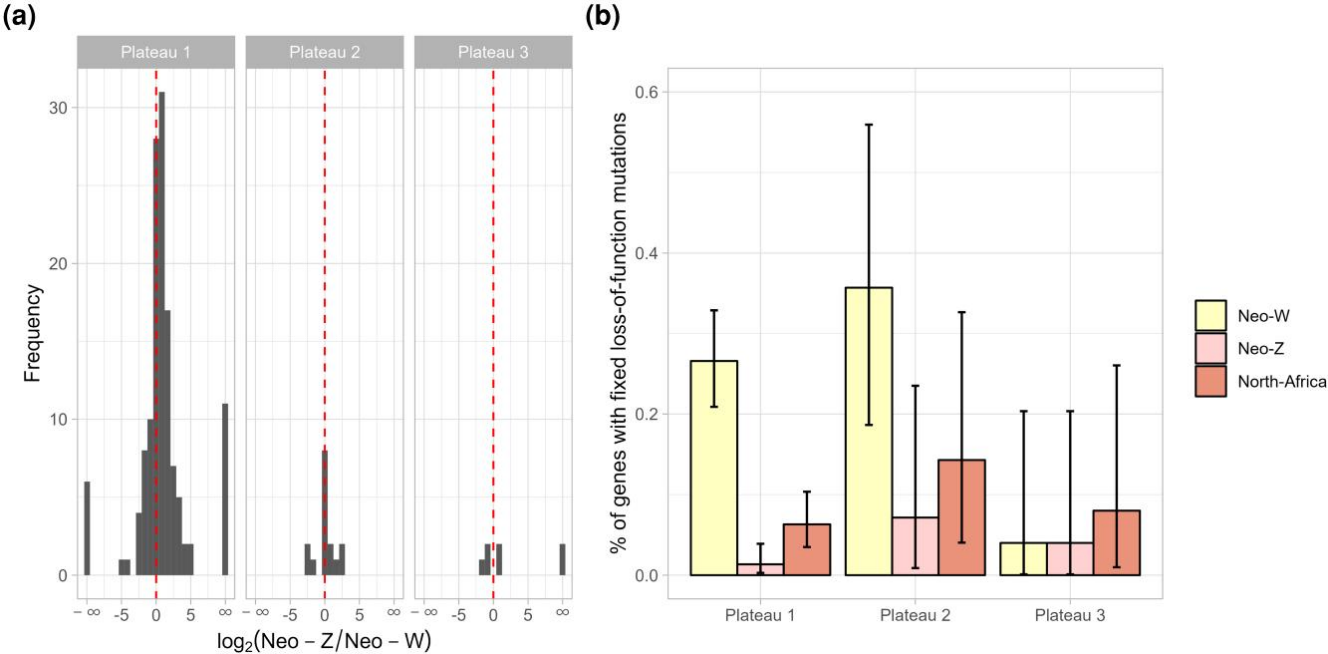
Neo-W chromosome degeneration. a) Gene expression of the neo-Z and neo-W chromosome, normalized by total expression (neo-Z + neo-W). b) The proportion of genes with fixed loss-of-function mutations on the neo-W is significantly greater for the oldest plateau (1) compared to both the neo-Z and the youngest plateau (3). Bars represent 95% binomial confidence intervals.

Given the reduced mutation load across plateau 2 and 3 (see next section and Fig. 6), we hypothesize that recombinant neo-W haplotypes were favored by natural selection. However, given the lack of recombination on the neo-W, the information about its selective history is limited and contained entirely within the neo-W genealogy (Fig. 5). In particular, it would be inappropriate to employ genome-scan methods for inferring selective sweeps. We find that the $T_{\mathrm{MRCA}}$ of the neo-W tree ($∼5\times 10^{4}$) is an order of magnitude younger than $T_{\mathrm{Plateau}\;3}$ ($5.0\times 10^{5}$). Thus, any signal of selective sweeps occurring $>5.0\times 10^{4}$ generations ago has since been lost.

### Neo-W Degeneration

The neo-W chromosome contains three plateaus that started degenerating at different time points, including one for which recombination arrest was recent (∼0.5 Mya). This provides an opportunity to investigate the temporal dynamics of sequence degeneration due to recombination suppression. Here, we quantify neo-W degeneration relative to homologous neo-Z sequence in terms of loss-of-function variants and gene expression.

We assessed sequence homology between the neo-Z and neo-W chromosome by comparing read depth in males and females. As expected, males were equally covered on the neo-sex chromosome and the autosomes (neo-sex:autosome depth ratio = 1). In contrast, the neo-sex chromosome in females showed reduced read depth (neo-sex:autosome depth ratio = 0.9) suggesting a mapping rate of 0.8 for neo-W reads compared to neo-Z reads. For plateau 1, neo-W chromosome reads had lower coverage than reads from the orthologous region of the Maghreb autosome (supplementary figs. S5 and S6, Supplementary Material online), despite having started diverging from the neo-Z at around the same time. This suggests that there has been sequence loss on the neo-W not experienced by the neo-Z or Maghreb autosome. By contrast, coverage did not significantly differ between the neo-Z and the neo-W on Plateau 3 (Wilcoxon rank-sum test, P>0.4), consistent with limited divergence and sequence loss in this region.

We next considered the frequency of deleterious variants on the neo-W, neo-Z, and the homologous Maghreb autosome. Consistent with substantial neo-W degeneration, 25% (95% binomial CI = 0.20 to 0.31) of genes contained loss-of-function mutations that were fixed on the neo-W, compared to 2% (0.008 to 0.05) on the neo-Z and 8% (0.04 to 0.11) on the Maghreb autosome. Degeneration of the neo-W differed between plateaus: 27% (0.21 to 0.33) of genes within plateau 1 contained fixed loss-of-function mutations, compared to only 4% (0.001 to 0.20) of genes on plateau 3 (Fig. 6). We also find that the neo-W has an elevated ratio of nucleotide diversity between 0D and 4D sites ($\pi_{0}/\pi_{4}=0.35$), compared to the neo-Z (0.26) and the Maghreb autosome (0.20). Elevated $\pi_{0}/\pi_{4}$ is consistent with a reduced efficacy of selection and an accumulation of weakly deleterious mutations at 0D sites.

We used previously published RNA-seq data from a single female (Mackintosh et al. 2019) to analyse haplotype-specific gene expression on the neo-sex chromosomes. We find that gene expression is biased towards the neo-Z, with a median fold-difference of 1.5 between the neo-Z and the neo-W (Fig. 6).

Overall, we find that the neo-W has lost DNA, and that premature stop-codons and frame-shift mutations have become fixed. Additionally, gene expression on the neo-W chromosome is reduced compared to the neo-Z.

## Discussion

### A Young Neo-Sex Chromosome System

Neo-sex chromosomes provide a means of investigating the evolutionary consequences of recombination arrest. Here, we have focused on a neo-sex chromosome system in Iberian *M. ines* butterflies, where a recent Z-autosome fusion has resulted in the homologous autosome becoming a nonrecombining neo-W chromosome. Our analysis of genome sequence data suggests that the neo-sex chromosomes originated $∼1.4\times 10^{6}$ generations ago. This neo-W is, therefore, older than the neo-Y of *D. albomicans*—the youngest neo-Y identified among *Drosophila* species (Zhou et al. 2012)—but younger than the neo-Y of *D. miranada* (Bachtrog and Charlesworth 2002). Similar to *Drosophila* neo-Y chromosomes, the *M. ines* neo-W shows evidence of degeneration. For example, 27% of genes contain loss-of-function mutations on plateau 1 of the neo-W. This level of degeneration is greater than that observed on the *D. albomicans* neo-Y chromosome (only 2% of genes contain loss-of-function mutations, Zhou et al. 2012), but similar to what is observed on the *D. miranda* neo-Y (Bachtrog 2005). Without a *de novo* assembled neo-W it is challenging to accurately quantify sequence degeneration, as many TE insertions or other deleterious structural variants are undetectable with mapping-based analyses. Nonetheless, the majority of genes on the neo-W lack loss-of-function mutations consistent with a young neo-sex chromosome system at an intermediate level of sequence degeneration.

### The Evolutionary History of the Neo-Sex Chromosomes

Our analysis suggests that the Z-autosome fusion and associated neo-sex chromosomes are restricted to the Iberian population of *M. ines*. While the Iberian Peninsula and the Maghreb were connected by land prior to the Zanclean flood ~5.3 Mya (Cornée et al. 2016), our demographic analyses suggest that the split between Iberian and North-African *M. ines* populations is much younger than the Strait of Gibraltar (1.5 Mya). This estimate of population divergence is based on intergenic sites, which do not evolve under strict neutrality in *M. ines* ($\pi_{4D}>\pi_{\mathrm{intergenic}}$) and—as a result—is likely an underestimate. Moreover, we calibrated the estimate with the mutation rate of *Heliconius melpomene*—whose most recent common ancestor with *M. ines* lived ∼60 to 80 Mya (Kumar et al. 2017). However, despite these caveats, the most likely scenario is that Iberian populations of *M. ines* were established long after the Zanclean flood.

Our estimates for the origin of the neo-sex chromosomes show that population and neo-sex chromosome divergence began at a similar time. However, given the overlapping confidence intervals of divergence times we are unable to infer a specific order for the two events. This task is especially difficult given that coalescent simulations do not yet accommodate population-specific recombination rates. Our parametric bootstrap instead assumed recombination on the neo-W, and, as a result, the estimated confidence interval for the neo-sex chromosome split is likely too narrow. Additionally, the comparison of split times assumes that the autosomes and sex chromosomes have equal mutation rates. While do not know if it is the case in *M. ines*, or Lepidoptera in general, male biased mutation rates have been observed in many organisms including *D. melanogaster* (Wang et al. 2023).

Although we have been unable to infer the full history of the neo-sex chromosomes, a plausible scenario is that the Iberian population was established from an ancestral North African population by migration, with the neo-sex chromosome arising and fixing shortly after in the isolated Iberian population. Interestingly, PSMC trajectories of $N_{e}$ change suggest no Iberian specific signal of an Out-of-Africa bottleneck (supplementary fig. S7, Supplementary Material online).

### Historical Recombination Events

**Fig. 7.**
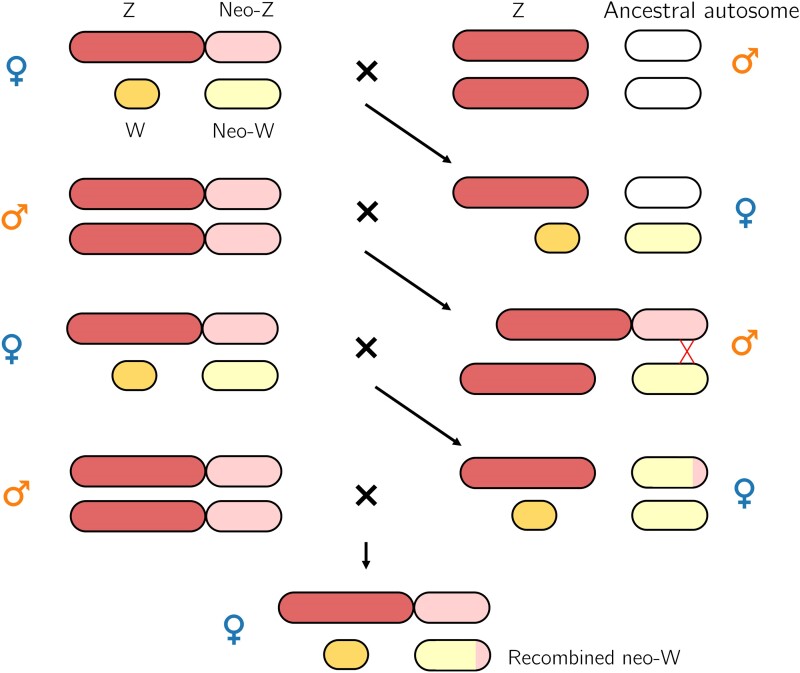
Neo-W escape in males. Mating between and an Iberian female with the derived Z-autosome fusion and a male with the ancestral, unfused arrangement can result in a female offspring with a W chromosome, a neo-W, a Z and an unfused autosome. In this female, the W and the neo-W are not linked. Consequently, the neo-W may be transmitted along with the Z chromosome. In males, the neo-W can then recombine with the neo-Z chromosome, before ultimately being coinherited with the W chromosome.

We find evidence for two historical recombination events between the neo-Z and the neo-W. One possibility is that recombination occurred in females. Rare spontaneous recombination despite achiasmy—both chiasma formation during meiosis and premeiotic ectopic recombination—has been observed in males of several *Drosophila* species: *D. melanogaster*, *D. simulans*, *D. virilis*, *inter alia* (Woodruff and Thompson 1977). Alternatively, rare migration into Iberia from populations with the ancestral (unfused) chromosome arrangement could have led to the neo-W migrating into males (Fig. 7). The F1 offspring of a migrant and an Iberian individual will include individuals with a W chromosome, a neo-W, a Z and an unfused autosome. In these F1s, the neo-W and the W are not linked, allowing the neo-W to be transferred to males by independent assortment. Recombination between the neo-Z and the neo-W could, therefore, have happened in heterokaryotypic males (Fig. 7). Although we do not find direct evidence for this scenario in *M. ines*, it is an expected outcome of rare migration events. A similar scenario has been reported in *Drosophila americana*; individuals with a derived X-autosome fusion reproduce frequently with those carrying the ancestral arrangement, thereby allowing recombination between the neo-X and the neo-Y in heterokaryotypic females (Charlesworth et al. 1997; McAllister 2003). While our results are compatible with rare crossover events in female Lepidoptera (which has never been detected), we stress that the population genomic signal of rare female recombination is indistinguishable from recombination in (presumably also rare) descendants of migrant individuals, i.e. we cannot discern between these two scenarios. How plausible the male escape hypothesis is depends on the strength of postzygotic and premating barriers between African and Iberian *M. ines* populations. Differences in wing patterns and habitat between populations on either side of the Strait of Gibraltar have led some taxonomists to consider North-African *M. ines* a distinct form (Wagner 1913).

The rare recombination between sex chromosomes in achiasmatic taxa we have found here can be viewed as the inverse of strata formation via rare recombination suppressors in classical models of sex chromosome evolution (in taxa where both sexes recombine) (Charlesworth 1978). We have, therefore, chosen the term “plateau” to highlight the distinction from strata. Our analysis of sequence degeneration suggests that the younger neo-W plateaus have reduced mutation load. This is expected given that the plateaus were formed by recombination with a neo-Z chromosome that was free from the load associated with recombination arrest. Following the recombination event, we would expect a recombinant neo-W haplotype to be under strong positive selection and to rapidly become fixed in the population. Importantly, there is a trade-off between selection against neo-W load and potential neo-W linked female beneficial mutations. Thus, a recombinant neo-W haplotype may not always be beneficial, because female-specific alleles could be lost. However, the reduced mutation load across the youngest plateau 3 (Fig. 6) gives reason to suspect that the recombinant neo-W haplotype swept through the population.

The neo-Z neo-W divergence time estimate for the third plateau is an estimate of the time of the sweep that established the last recombinant neo-W haplotype in the Iberian *M. ines* population. Conversely, the $T_{\mathrm{MRCA}}$ of the neo-W genealogy is a lower bound of the sweep time. However, since the neo-W $T_{\mathrm{MRCA}}$ is an order of magnitude younger than the divergence of the third plateau, neo-W diversity cannot contain any information about the sweep associated with the third plateau. This is true even if one were to assume an extreme null model which assumes that neo-W diversity evolves neutrally following the sweep (i.e. no background selection) and so recovers to one-third of that of the neo-Z. The strongly reduced genetic diversity on the neo-W (and our corresponding estimates of $N_{W}$) suggest that—unsurprisingly—other strong selection must have been acting on the neo-W. Importantly, this will include selection on the mitochondrion, the ancestral W and any maternally inherited endosymbiont, given the maternal inheritance of the neo-W and its complete linkage to these other chromosomes. We find no evidence of any recent recombination affecting current neo-W diversity. While the fact that neo-W diversity can be placed on a single genealogy (Fig. 3) simplifies analysis, it also means that there is limited power to make detailed inferences about the population processes that generated the neo-W genealogy.

Our finding of rare recombination between neo-sex chromosomes shows that sequence degeneration following recombination arrest can be reversible, and mirrors previous results in certain *Drosophila* species (Charlesworth et al. 1997; McAllister 2003; Wei and Bachtrog 2019). As more neo-sex chromosomes are being reported in Lepidoptera (Martin et al. 2020; Mackintosh et al. 2022; Höök et al. 2023; Rueda-M et al. 2023), it will be interesting to see whether the neo-W degeneration and recombination observed in *M. ines* is representative of neo-sex chromosome evolution in this order.

## Materials and Methods

### Sampling and Sequencing

Fifteen butterflies were collected in Iberia and the Maghreb (supplementary table S1, Supplementary Material online). A high molecular weight (HMW) DNA extraction was performed for the genomic reference sample PT_MI_8 (male) using a salting out method (see Mackintosh et al. 2022 for details). For the other 14 samples, DNA was extracted from thoracic tissue with a Qiagen DNeasy Blood & Tissue kit. DNA libraries were prepared with the Illumina Truseq Nano kit. The paired-end libraries were sequenced on an Illumina NovaSeq6000 machine. A Pacbio CLR library was prepared from the HMW DNA and sequenced on a Sequel I machine. Additionally, an Arima HiC reaction was performed with flash frozen thoracic tissue from the female sample PT_MI_86. A TruSeq library was prepared from the crosslinked DNA and sequenced on an Illumina NovaSeq 6000.

#### Genome Assembly and Haplotype-Specific HiC Maps

We generated a reference genome for *M. ines* by assembling Pacbio continuous long reads with NextDenovo version 2.4.0 (Hu et al. 2023). The contig sequences were polished with Illumina short-reads from the same individual (PT_MI_8) using Hapo-G version 1.1 (Aury and Istace 2021). We identified and removed haplotypic duplicates and contigs deriving from other organisms using purge_dups version 1.2.5 (Guan et al. 2020) and blobtools version 1.1.1 (Laetsch and Blaxter 2017), respectively. We mapped HiC data (from PT_MI_86) to the contigs with bwa-mem version 0.7.17 (Li and Durbin 2009) and then used YaHS version 1.1a.2 and juicebox version 1.11.08 to scaffold the assembly into chromosome-level sequences (Robinson et al. 2018; Zhou et al. 2023). We also generated haplotype-specific HiC maps following Mackintosh et al. (2022) to further investigate the karyotype of the female individual used for HiC sequencing (PT_MI_86).

#### Gene Annotation

Repetitive elements in the genome assembly were masked with Red (Girgis 2015). Two previously published RNA-seq datasets (Mackintosh et al. 2019) were aligned to the assembly with HISAT2 2.1.0 (Kim et al. 2019). The repeat masked genome assembly and RNA-seq alignments were used to annotate genes with braker2.1.5 (Stanke et al. 2006, 2008; Li et al. 2009; Barnett et al. 2011; Lomsadze et al. 2014; Buchfink et al. 2015; Hoff et al. 2015, 2019).

#### Variant Calling and Filtering

WGS reads were trimmed with fastp 0.20.0 (Chen et al. 2018) and aligned to the reference genome using bwa-mem (bwa 0.7.17; Li and Durbin 2009). Duplicate reads were marked with Sambamba 0.6.6 (Tarasov et al. 2015). Freebayes v1.3.2-dirty (Garrison and Marth 2012) was used to call variants.

We used the gIMble prep module (Laetsch et al. 2023) to filter variants: variants were normalized and decomposed with bcftools 1.12 (Danecek et al. 2021) and vcfallelicprimitives (Garrison et al. 2022), respectively. Single nucleotide polymorphisms (SNPs) with support from a single strand, or from unbalanced reads (present solely on the right or the left side of the alternate allele) or within 2 bp of non-SNP variants were excluded. Only SNPs with a minimum genotype quality of 10 and read depth between 8 and 3 × mean genome depth were retained. Callable regions of the genomes—i.e. with read depth between 8 and 3 × mean genome coverage—were identified using mosdepth 0.3.2 (Pedersen and Quinlan 2018). Excluded SNPs were removed from callable regions. A VCF containing indels was produced by filtering variants as described above, with the exception that indels were retained.

### Estimation of Diversity and Divergence

Mean and windowed estimates of genetic diversity (*π* and *H*), divergence ($d_{xy}$), and differentiation ($F_{\mathrm{ST}}$ and $d_{a}$) were computed with custom-made python scripts (see Data accessibility). Coding sites—4-fold (4D) and 0-fold (0D)—were classified with codingSiteTypes.py available at: https://github.com/simonhmartin/genomics_general. The neo-W chromosome has regions that map poorly to the neo-Z reference assembly leading to biased estimates of divergence. When computing neo-W neo-Z divergence we, therefore, removed the 20% of windows with the lowest neo-W coverage, as the neo-W was ∼80% covered. Bayesian change point detection for windowed estimates of diversity and divergence was conducted with the bcp package (Erdman and Emerson 2007).

### Neo-Sex Chromosome Detection

The genetic structure of the neo-sex chromosome and the autosomes were characterized with (i) principal component analyses and (ii) phylogenetic tree constructions. For PCAs, eigenvectors and eigenvalues of the genotype matrices were computed with Scikit-allel v1.3.5 (Miles et al. 2023). For phylogenetic trees, IUPAC consensus sequences were created for each individual using bcftools with the consensus command. Consensus sequences were aligned with MAFFT v7.520 (Katoh et al. 2002) and trimmed with trimAl v1.4 (Capella-Gutiérrez et al. 2009). Maximum likelihood phylogenetic trees were inferred from trimmed alignments with IQ-TREE 2 (Minh et al. 2020).

### Neo-Sex Chromosome Phasing

Partial phasing of variants on the neo-sex chromosomes was achieved by identifying neo-W-specific alleles and extracting reads containing such alleles, as devised by Martin et al. (2020). We first identified neo-W specific alleles as alternative alleles that are present in a single copy in each Iberian female but absent in all Iberian males using a custom python script. Aligned WGS reads containing neo-W-specific alleles were isolated using an adapted version of the script filterSAMbyTargetBase.py available at: https://github.com/simonhmartin/genomics_general. Variants were called with freebayes v1.3.2 and filtered using gIMble prep with the aforementioned parameters (except with the minimum read depth relaxed to 3, given the haploid nature of neo-W chromosomes).

### Demographic History Inference

Demographic inference was performed with gIMble (Laetsch et al. 2023) which fits a model of isolation with migration assuming an ancestral population that splits into two derived populations at time *T* followed by unidirectional gene flow from one population to the other at rate $m_{e}$. The effective population size ($N_{e}$) of the ancestral and derived populations are allowed to differ. Two nested models assuming either strict divergence (DIV, $m_{e}=0$) or long-term migration (MIG, T→∞) can also be fitted with gIMble.

We fitted DIV, IM, and MIG models to autosomal sequence of *M. ines* to infer the demographic history of the Iberian and Eastern Maghreb populations. To infer the history of the neo-sex chromosome, we created pseudo-diploid neo-W data (by combining phased neo-W haplotypes). We fitted a DIV model between the neo-W pseudo-diploids and Iberian males (i.e. neo-Z diploids) with gIMble. In both cases, the analysis was restricted to intergenic sites, with a block size of 64 bp. We assumed a mutation rate of $2.9\times 10^{-9}$ mutations per site per generation, the estimated rate for *H. melpomene* (Keightley et al. 2015). We assumed a generation time of 1 year as *M. ines* is univoltine.

### Neo-Sex Chromosome Coverage

Mean read depth per base (coverage) was evaluated with mosdepth 0.3.2 (Pedersen and Quinlan 2018). We used the difference in coverage between males (neo-Z + neo-Z) and females (neo-W + neo-Z) to estimate the coverage of neo-W chromosomes relative to neo-Z chromosomes. More specifically, we estimated neo-W coverage as ${\mathrm{cov}}_{W}=({\mathrm{cov}}_{\mathrm{ZW}}-{\mathrm{cov}}_{\mathrm{ZZ}}/2)\times 2=({\mathrm{cov}}_{\mathrm{Female}}-{\mathrm{cov}}_{\mathrm{Male}}/2)\times 2$.

### Gene Expression Analysis

We used previously published RNA-seq data from a female (PT_MI_61) (Mackintosh et al. 2019) to analyse gene expression on the neo-sex chromosome. In order to obtain haplotype-specific gene expression profiles on the neo-sex chromosome of the female PT_MI_61, we identified neo-W and neo-Z diagnostic alleles from the WGS dataset. To minimize reference bias, we also created a pseudo-reference assembly with neo-W alleles from PT_MI_61 (as in Wei and Bachtrog 2019). We then mapped the trimmed RNA-seq reads to both the reference assembly and pseudo-reference with HISAT2 2.1.0 (Kim et al. 2019). Neo-Z specific RNA-seq reads were extracted from the alignments to the reference assembly and neo-W specific RNA-seq reads were extracted from alignments to the pseudo-reference, as described for the WGS reads. Gene expression was subsequently quantified with HTseq-count 2.0.2 (Anders et al. 2015).

### Nonfunctional Genes

Loss-of-function mutations, here defined as premature stop codons, frame-shift mutations, stop-loss and start-loss variants, were detected with SnpEff 5.1d (Cingolani et al. 2012) from the VCF containing both SNP and indel calls. We estimated the proportion of genes with fixed derived loss-of-function mutations on the neo-W, the neo-Z and the African homolog. We only considered genes with nonzero expression in the Iberian female PT_MI_61, i.e. which were expressed either by the neo-Z or the neo-W chromosome. This excluded spurious genes from the analysis, as well as genes which could not be phased.

### Tree Test

Given that the neo-W is a nonrecombining chromosome, genetic variation among these sequences should reflect a single genealogy. For a single neo-W tree with 7 tips, we expect 11-folded variant types. By contrast, we expect recombining sequences, such as the neo-Z, to show evidence of all 63 $(\sum_{i=1}^{\left\lfloor n/2\right\rfloor }(\begin{array}{c} n\\ i \end{array}))$ possible branches types (Felsenstein 1978). With this in mind, we summarized the neo-W and neo-Z haplotype alignments by extracting and counting SNP types and compared them to a neutral expectation.

We used the frequency of neo-W SNP types to construct an unrooted maximum parsimony tree. We estimated the root node age of the neo-W tree (time to the most common ancestor, $T_{\mathrm{MRCA}}$) as the average proportion of derived mutations (excluding fixed derived sites) per individual divided by the mutation rate. We polarized neo-W alleles with North-African alleles, using a custom python script. SNPs that were not consistent with the neo-W tree were discarded, whereas those which were consistent with the single tree but mispolarized (e.g. [0, 0, 0, 1] mispolarized as [1, 1, 1, 0]), were reassigned to the correct SNP type.

## Supplementary Material

msae124_Supplementary_Data

## Data Accessibility

All the scripts and commands used for this project are available at: https://github.com/thomdec/neosex_melanargia. The *Melanargia ines* genome assembly and all raw sequencing data are available at the European Nucleotide Archive under project accession PRJEB71083. The gene annotation, repeat intervals, callable intervals, and VCF file are available within a Dryad repository https://doi.org/10.5061/dryad.fj6q57435.
