# Supplementary material for: Rewinding the Ratchet: Rare Recombination Locally Rescues Neo-W Degeneration and Generates Plateaus of Sex-Chromosome Divergence
Source: Mol Biol Evol. 2024 Jul 1;41(7):msae124. doi: 10.1093/molbev/msae124 (PMC11232697; doi:10.1093/molbev/msae124)
Supplement: msae124_Supplementary_Data [file msae124_supplementary_data.zip › Melanargia_Neosex_Manuscript_revised_supp.pdf]

Supplementary materials

Supplementary text

Phasing

To detect loss-of-function mutations on the neo-W and neo-Z chromosomes, we first phased the neo-sex chromosomes in females. Partial phasing of the neo-sex chromosome was achieved by identifying and extracting neoZ- and neo-W-specific reads. Neo-W diagnostic alleles were defined as alternative alleles present in single copy in all seven females, while absent in males, at sites where all females are heterozygous. On the neo-sex chromosome, 69% of sites heterozygous across all females had neo-W specific alleles. One might expect alleles that are present in a single copy in all seven females, and absent in all three males, purely by chance. However, this distribution of genotypes is extremely rare on other autosomes: only 0.4% of sites heterozygous across all females on the autosomes. This negligible error rate suggest that robustness of our simple phasing method. On average, 36% of female reads mapping to the reference neo-W were classified as neo-W reads. Since female had an average read depth of 90%, the neo-W was expected to account for 40% of the reads on the neo-sex chromosome, assuming that the neo-Z was fully covered. We thus estimate that $0.36/0.40 = 90\%$ of the neo-W chromosome was successfully phased.

Parametric bootstrap

We estimated confidence intervals around the inferred split time T by parametric bootstrap. We generated bootstrap replicates of the best-fitting model with the gIMble wrapper for msprime (Baumdicker et al. 2021), and computed the maximum composite likelihoods (MCLEs) parameters of the replicates. For the origin of the neo-sex chromosome, we simulated 100 bootstrap replicates under the best-fitting model and fitted the replicates to a DIV model. We assumed a recombination rate of 1.9 cM/Mb, i.e. one crossover per tetrad per male meiosis for the neo-sex chromosome, assuming 50% female in the population ($\frac{100 \text{ cM} \times 1/4}{13 \text{ Mb}} = 1.9$ cM/Mb). Idem for the autosomal demographic history of *M. ines*, except with a recombination rate of 0.7 cM/Mb — the weighted mean of autosome lengths (34 Mb) is greater than the size of the neo-sex chromosome (13 Mb), explaining the lower recombination rate for autosomal regions.

Table S1 – Sequenced individual metadata

Sample	Preservation	Species	Sex	Country	Latitude	Longitude	Data
PT_MI_7	Liquid nitrogen	*Melanargia ines*	M	Portugal	37.67	-7.738	WGS
PT_MI_8	Liquid nitrogen	*Melanargia ines*	M	Portugal	37.67	-7.738	WGS, Pacbio
PT_MI_61	Liquid nitrogen	*Melanargia ines*	F	Portugal	37.211	-7.438	WGS
PT_MI_86	Liquid nitrogen	*Melanargia ines*	F	Portugal	37.411	-7.802	WGS, Hi-C
TN_MI_1619	Ethanol	*Melanargia ines*	F	Tunisia	35.876	8.547	WGS
MA_MI_1620	Ethanol	*Melanargia ines*	M	Morocco	30.61	-7.58	WGS
DZ_MI_1624	Ethanol	*Melanargia ines*	M	Algeria	36.004	4.227	WGS
ES_MI_1647	Ethanol	*Melanargia ines*	M	Spain	39.875	-0.417	WGS
ES_MI_1680	Ethanol	*Melanargia ines*	F	Spain	39.93	-4.474	WGS
DZ_MI_1681	Ethanol	*Melanargia ines*	M	Algeria	35.1	1.15	WGS
ES_MI_1682	Ethanol	*Melanargia ines*	F	Spain	42.12639	-1.39389	WGS
ES_MI_1683	Ethanol	*Melanargia ines*	F	Spain	39.60081	-7.18825	WGS
ES_MI_1684	Ethanol	*Melanargia ines*	F	Spain	38.01498	-1.37461	WGS
DZ_MI_1685	Ethanol	*Melanargia ines*	F	Algeria	36.00083	4.297778	WGS
ES_MI_1686	Ethanol	*Melanargia ines*	F	Spain	36.8426	-5.35515	WGS

Table S2 – The number of HiC read pairs mapping exclusively to each chromosome. Values are shown for the entire HiC dataset as well as for the haplotype-specific datasets. The sequenced individual was female, so possesses a single Z chromosome (13) along with neo-Z and neo-W chromosomes (14).

Chromosome	Total (pairs per-kb)	Haplotype 1 (prop. of total)	Haplotype 2 (prop. of total)
1	3,345,097 (65)	230,617 (.069)	231,021 (.069)
2	2,572,798 (64)	177,877 (.069)	177,986 (.069)
3	2,437,107 (64)	154,822 (.064)	155,530 (.064)
4	2,311,788 (64)	151,446 (.066)	151,316 (.065)
5	2,175,740 (64)	135,816 (.062)	137,844 (.063)
6	2,031,116 (65)	146,566 (.072)	148,012 (.073)
7	1,984,385 (64)	134,555 (.068)	135,150 (.068)
8	1,716,662 (63)	111,829 (.065)	111,807 (.065)
9	1,685,865 (64)	105,027 (.062)	106,176 (.063)
10	1,541,564 (61)	97,885 (.063)	98,403 (.064)
11	1,473,702 (59)	97,064 (.066)	98,162 (.067)
12	1,460,150 (65)	103,743 (.071)	104,704 (.072)
13 (Z)	698,026 (36)	1,435 (.002)	2,515 (.004)
14 (neo-Z, neo-W)	612,340 (47)	35,503 (.058)	97,439 (.159)

Table S3 – Read depth on the Z and neo-Z chromosomes, relative to the autosomes.

id	Z:Autosome	Neo-Z:Autosome
DZ_MI_1624	1.036	0.982
DZ_MI_1681	1.056	0.960
DZ_MI_1685	0.531	1.009
ES_MI_1647	1.005	0.984
ES_MI_1680	0.482	0.876
ES_MI_1682	0.524	0.905
ES_MI_1683	0.500	0.909
ES_MI_1684	0.501	0.896
ES_MI_1686	0.524	0.905
MA_MI_1620	1.059	1.000
PT_MI_7	1.007	1.007
PT_MI_8	1.019	1.000
PT_MI_61	0.505	0.897
PT_MI_86	0.500	0.917
TN_MI_1619	0.535	0.950

Table S4 – Model fit. $\Delta \ln \mathrm{CL}$ measured relative to the best fitting model, DIV

Model	DIV	$IM_{\rightarrow \mathrm{EM}}$	$IM_{\rightarrow \mathrm{IP}}$	$MIG_{\rightarrow \mathrm{EM}}$	$MIG_{\rightarrow \mathrm{IP}}$
$\Delta \ln \mathrm{CL}$	0	0	0	-5,607,055	-4,004,366

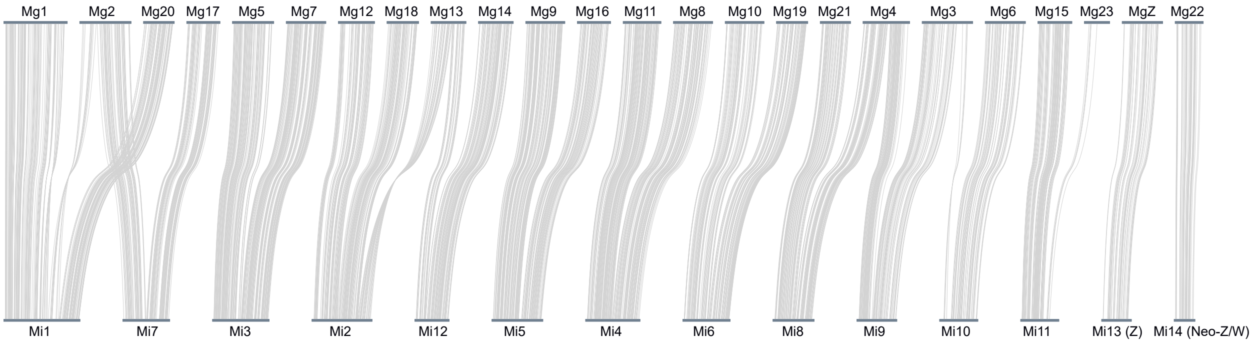

Figure S1 – Minimap alignment between *M. ines* and *M. galathea* assemblies

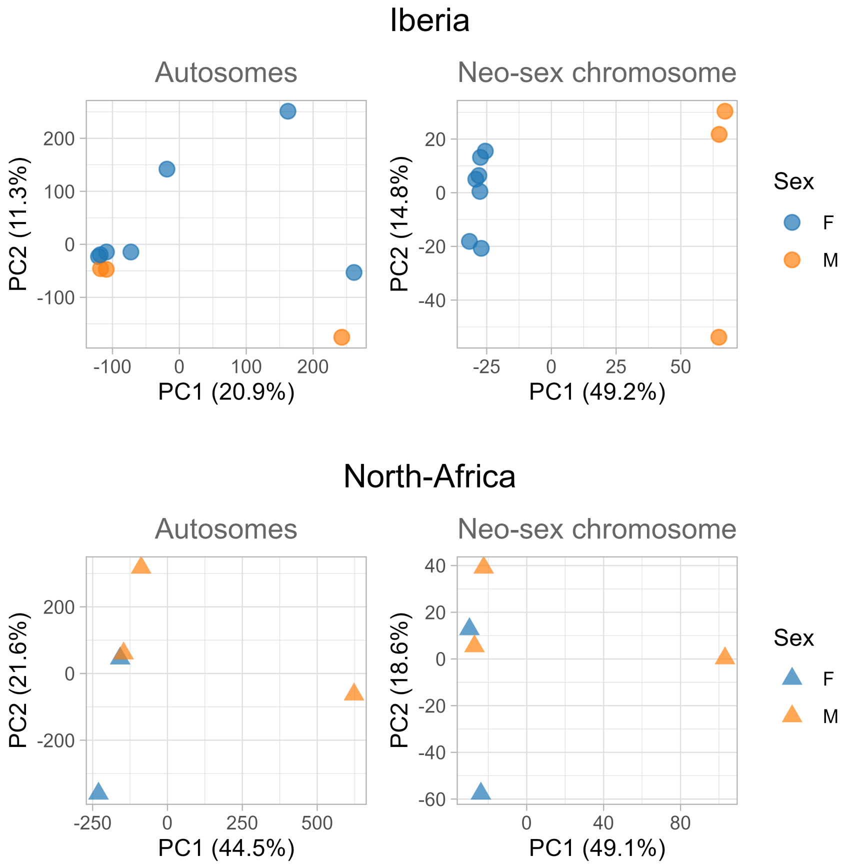

Figure S2 – Detection of the neo-sex chromosome. PCA for the autosomes and chromosome 14, for samples from the Iberian peninsula and the Maghreb. Chromosome 14 clusters by sex for sample from the Iberian peninsula. By contrast, PCA on autosomes and chromosome 14 show similar patterns

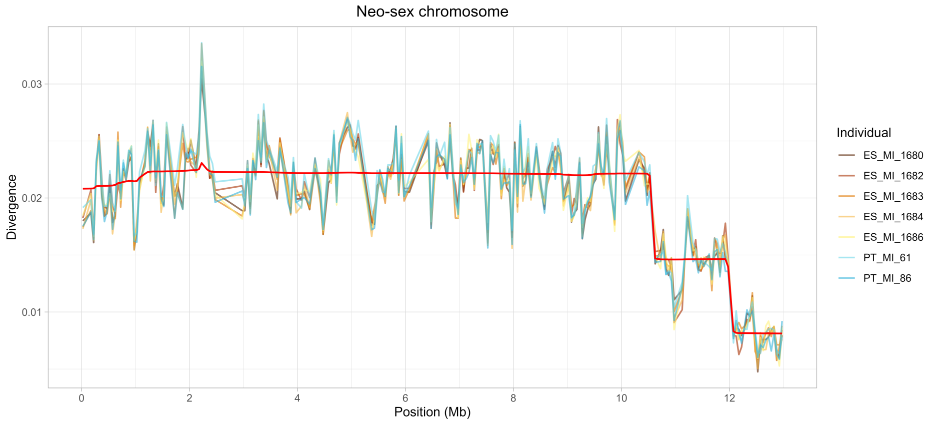

Figure S3 – Per-site heterozygosity on the neo-sex chromosome for Iberian females. The red line represents the posterior mean of divergence between the neo-Z and neo-W chromosomes.

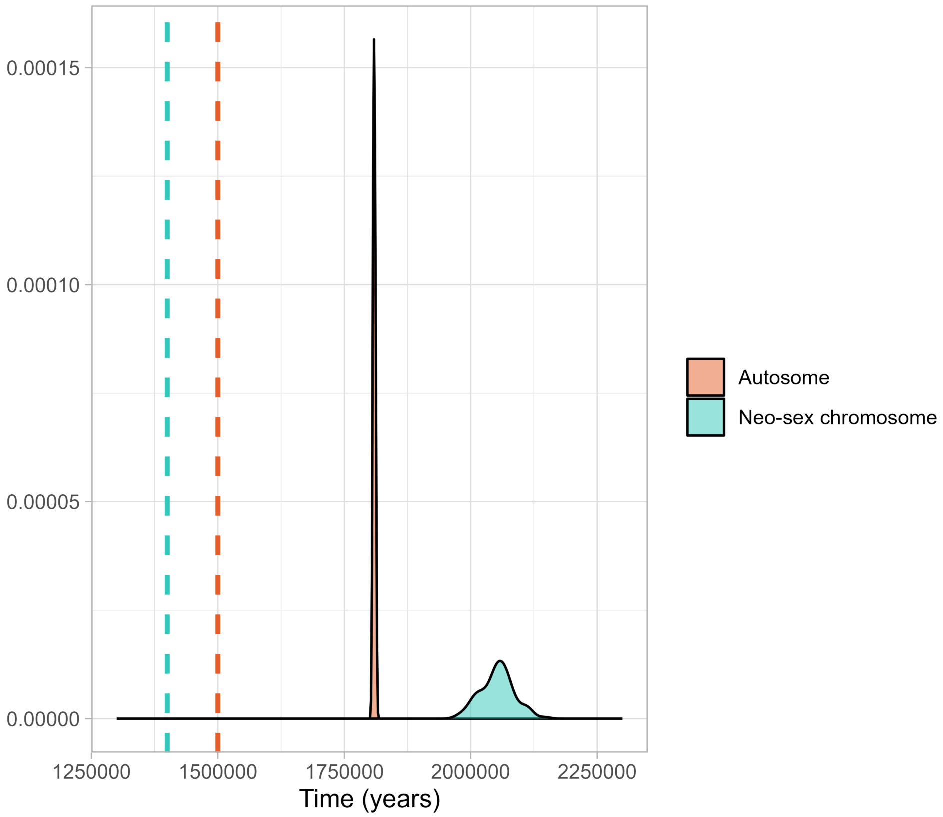

Figure S4 – Density of maximum composite likelihood estimate for bootstrap replicates. Dashed lines are the maximum composite likelihood estimates of the original data

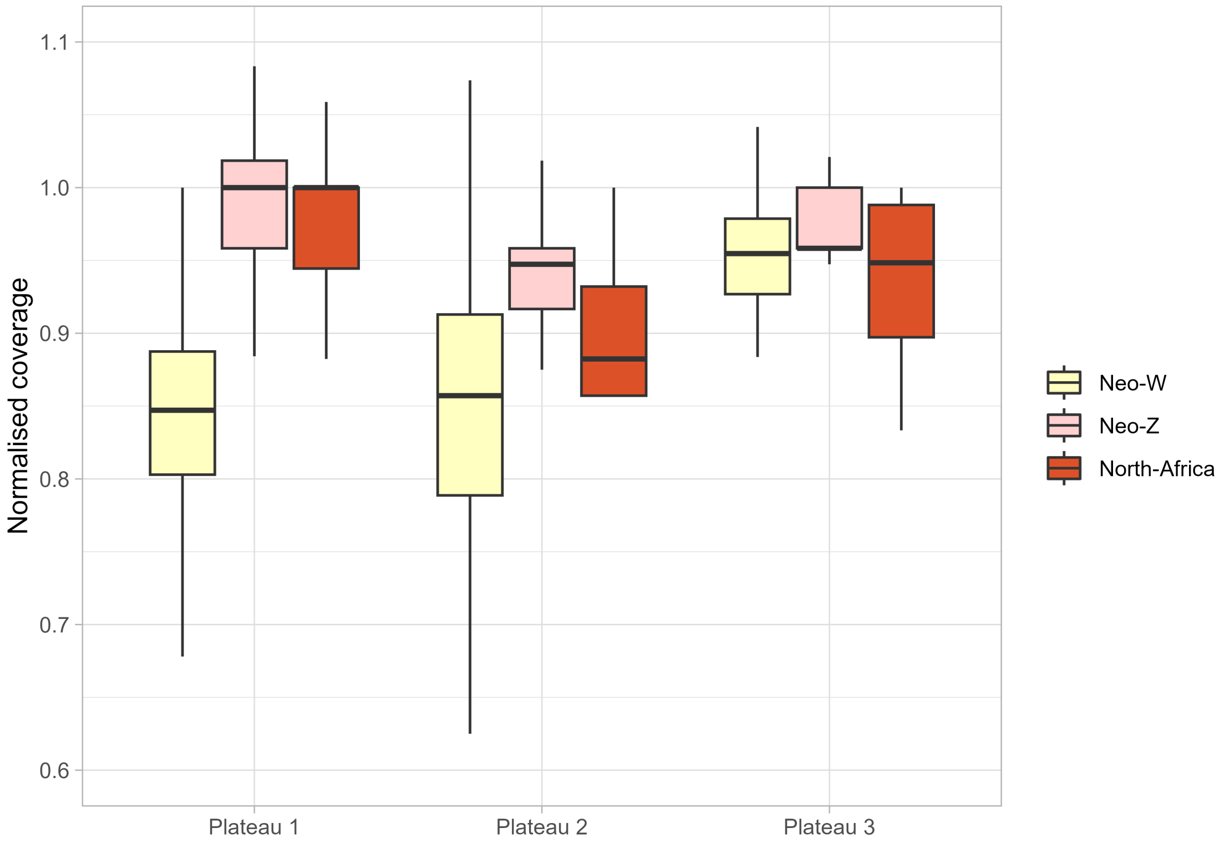

Figure S5 – Relative coverage for the neo-Z and neo-W chromosomes, and their North-African homolog. Read depth was computed in windows of 100kb and normalised against the median autosomal coverage.

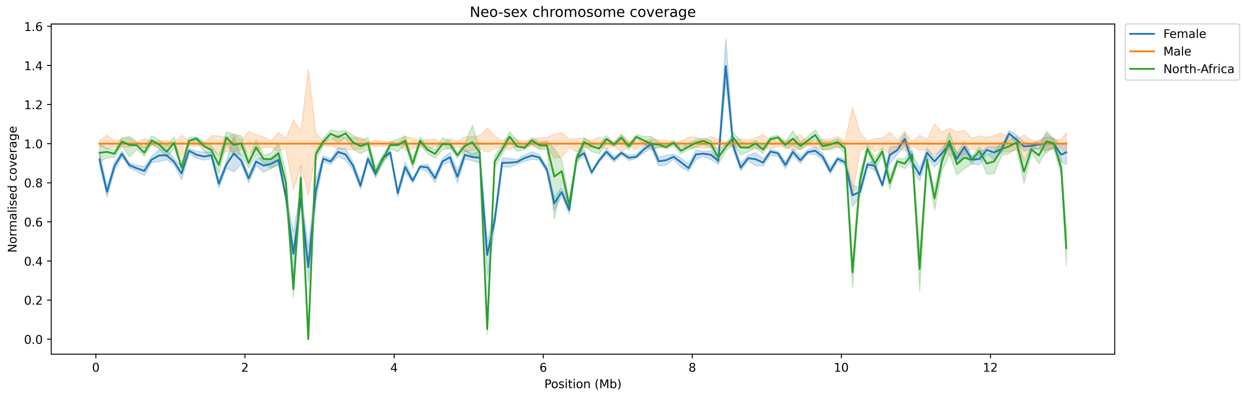

Figure S6 – Relative coverage along the neo-sex chromosome for Iberian males and females, and the north-african autosomal homolog. Read depth was computed in windows of 100kb and normalised against the median autosomal coverage.

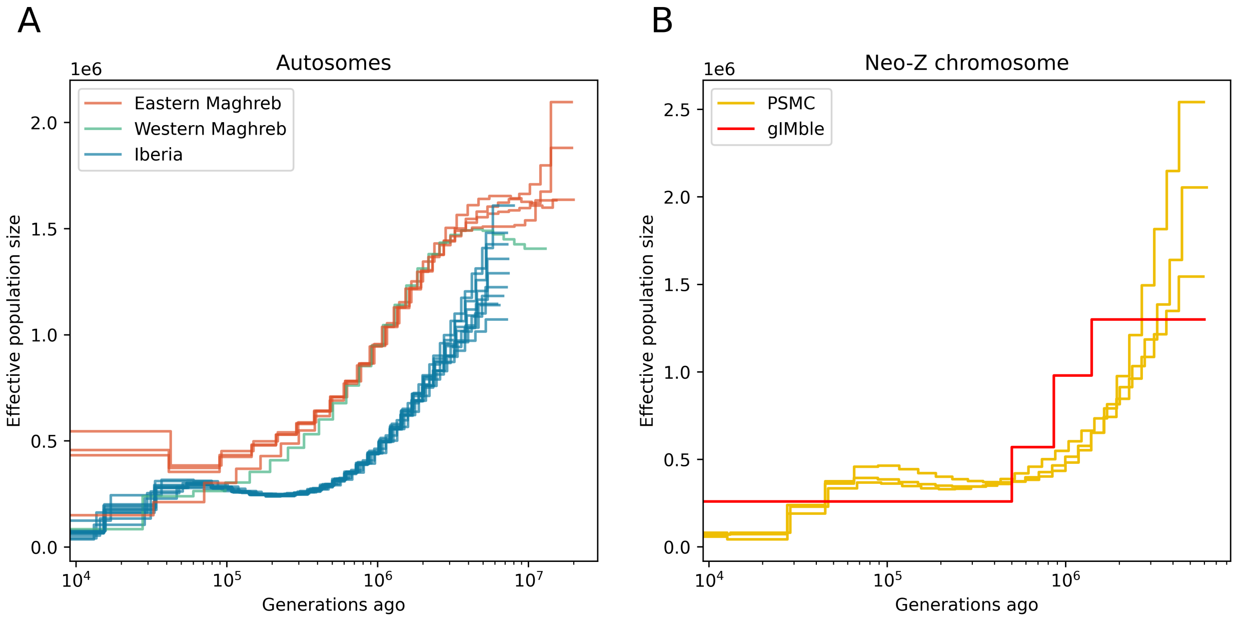

Figure S7 – A) PSMC-inferred demographic history of samples from Iberia, Eastern and Western Maghreb. The three populations show similar patterns. B) Demographic history of the neo-Z chromosome, as inferred by PSMC for three Iberian males, and by gIMble using the ancestral population size and split times inferred for each plateaus. Reassuringly, the two inferred trajectories showed a close fit
